# Prevalence and trends of metabolic syndrome among adults in the asia-pacific region: a systematic review

**DOI:** 10.1186/s12889-017-4041-1

**Published:** 2017-01-21

**Authors:** P. Ranasinghe, Y. Mathangasinghe, R. Jayawardena, A. P. Hills, A. Misra

**Affiliations:** 1 Department of Pharmacology, Faculty of Medicine, University of Colombo, Colombo, Sri Lanka; 2 Department of Physiology, Faculty of Medicine, University of Colombo, Colombo, Sri Lanka; 3 Institute of Health and Biomedical Innovation, Queensland University of Technology, Brisbane, Queensland Australia; 4 School of Health Sciences, Faculty of Health, University of Tasmania, Tasmania, Australia; 5 Fortis-C-DOC Centre of Excellence for Diabetes, Metabolic Diseases and Endocrinology, Chirag Enclave, New Delhi, India

**Keywords:** Metabolic syndrome, Prevalence, Trends, Asia-Pacific

## Abstract

**Background:**

The Asia-Pacific region is home to nearly half of the world’s population. The region has seen a recent rapid increase in the prevalence of obesity, type-2 diabetes and cardiovascular disease. The present systematic review summarizes the recent prevalence and trends of Metabolic Syndrome (MetS) among adults in countries of the Asia-Pacific Region.

**Methods:**

Data on MetS in Asia-Pacific countries were obtained using a stepwise process by searching the online Medline database using MeSH terms ‘Metabolic Syndrome X’ and ‘Epidemiology/EP’. For the purpose of describing prevalence data for the individual countries, studies that were most recent, nationally representative or with the largest sample size were included. When evaluating secular trends in prevalence in a country we only considered studies that evaluated the temporal change in prevalence between similar populations, prospective studies based on the same population or National surveys conducted during different time periods.

**Results:**

This literature search yielded a total of 757 articles, and five additional article were identified by screening of reference lists. From this total, 18 studies were eligible to be included in the final analysis. Of the 51 Asia-Pacific countries (WHO) we only located data for 15. There was wide between country variation in prevalence of MetS. A national survey from Philippines conducted in 2003 revealed the lowest reported prevalence of 11.9% according to NCEP ATP III criteria. In contrast, the highest recorded prevalence in the region (49.0%) came from a study conducted in urban Pakistan (Karachchi, 2004). Most studies reported a higher prevalence of MetS in females and urban residents. Data on secular trends were available for China, South Korea and Taiwan. An increase in the prevalence of MetS was observed in all three countries.

**Conclusion:**

Despite differences in methodology, diagnostic criteria and age of subjects studied, the Asia-Pacific region is facing a significant epidemic of MetS. In most countries nearly 1/5th of the adult population or more were affected by MetS with a secular increase in prevalence. Strategies aimed at primary prevention are required to ameliorate a further increase in the epidemic and for the reduction of the morbidity and mortality associated with MetS.

**Electronic supplementary material:**

The online version of this article (doi:10.1186/s12889-017-4041-1) contains supplementary material, which is available to authorized users.

## Background

Since being first described by Gerald M. Raevan in the 1980s numerous definitions and diagnostic criteria have been coined for Syndrome X (also known as metabolic syndrome) [1]. The International Diabetes Federation (IDF), National Cholesterol Education Program Adult Treatment Panel – III (NCEP ATP-III) guidelines and many others have defined metabolic syndrome (MetS) as a cluster of inter-connected metabolic abnormalities involving glucose metabolism (diabetes mellitus), lipid metabolism (hypercholesterolaemia and dyslipidaemia), elevated blood pressure and central obesity [2]. MetS increases the risk of type-2 diabetes, cardiovascular disease and all cause mortality [3]. It is also related to other co-morbidities including, pro-thrombotic and pro-inflammatory states, non-alcoholic steatohepatitis and reproductive disorders. Furthermore, the association of MetS with certain types of cancers is increasingly described in the literature [4].

The pathophysiology of MetS is complex with insulin resistance and abnormal regulation of lipid metabolism playing a central role in pathogenesis [5, 6]. Genetic predisposition is a factor in MetS, and prevalence differs among ethnic groups [7]. Some studies have demonstrated heritability of up to 70% involving HDL genes [8]. Age, lifestyle factors and socioeconomic status also play a major role in the pathogenesis [3]. Studies assessing the prevalence of MetS report conflicting results due to variations in diagnostic criteria. However, irrespective of the criteria used it is well accepted that the prevalence of MetS is increasing in epidemic proportions in both developed and developing countries globally [3]. The worldwide prevalence of MetS in the adult population is estimated to be 20–25% [9]. This rapid increase in MetS has been paralleled by the growing epidemic of type-2 diabetes, hypertension, cardiovascular disease and obesity [10].

The Asia-Pacific region typically includes much of Southeast Asia, and Oceania [11]. Countries in the region have a wide diversity in socio-cultural background and are at different levels of economic and technological development. Increasing economic development in many of the lower to middle-income countries of the region has been a major contributor to the increasing prevalence of obesity, type-2 diabetes and cardiovascular disease [12–14]. Hence, it is likely that the prevalence of MetS has also increased in the region in recent years. Several systematic reviews and meta-analyses on the prevalence of MetS have been published on South and East Asian populations [15]. However to date no studies have evaluated the prevalence of MetS in the Asia-Pacific region. Identification of the regional disease burden and trends will enable the prioritization and implementation of interventions through existing regional collaborations. The present systematic review summarizes the recent prevalence and trends of MetS among adults in countries of the Asia-Pacific Region.

## Methods

The systematic review was performed following the Preferred Reporting Items for Systematic Reviews and Meta-Analyses (PRISMA) guidelines and the PRISMA checklist is provided as a (Additional file 1).

### Search strategy

Data on MetS in Asia-Pacific countries were obtained using a stepwise process. We began our literature review by searching the online Medline database (Medical Literature Analysis and Retrieval System) using MeSH (Medical Subject Heading) term ‘Metabolic Syndrome X’ as a MeSH major topic and Epidemiology/EP as a MeSH subheading. The search comprised studies listed up to 30^th^ of April 2016. The search limits were; language (‘English’), Species (‘Humans’) and age (‘all adults: 19+ years’). The conjunction of the above results were narrowed down by including the names of the individual Asia-Pacific countries as defined by the World Health Organization (Afghanistan, American Samoa, Australia, Bangladesh, Bhutan, Brunei Darussalam, Cambodia, China, Cook Islands, Democratic People's Republic of Korea, Fiji, French Polynesia, Guam, Hong Kong, India, Indonesia, Japan, Kiribati, Lao, Macau, Malaysia, Maldives, Marshall Islands, Micronesia, Mongolia, Myanmar, Nauru, Nepal, New Caledonia, New Zealand, Niue, Northern Mariana Islands, Pakistan, Palau, Papua New Guinea, Philippines, Pitcairn Islands, Republic of Korea, Samoa, Singapore, Solomon Islands, Sri Lanka, Taiwan, Thailand, Timor-Leste, Tokelau, Tonga, Tuvalu, Vanuatu, Vietnam and, Wallis and Futuna) [16]. In stage two the total number of articles obtained from searching the Medline database utilizing the search criteria defined above, were screened for suitability for inclusion by reading the article ‘title’ and ‘abstract’. The studies not meeting with inclusion criteria (defined below) were removed during stage two. The remaining studies were further screened for suitability during the third stage by reading the full-text of the selected manuscript. To obtain additional data, a manual search was performed using the reference lists of selected articles. This process was conducted by two independent reviewers (PR and YM) and the final group of articles to be included in the review was determined after an iterative consensus process among the reviewers.

### Inclusion/Exclusion criteria and definitions

The following inclusion criteria were applied: a) population-based studies among healthy non-institutionalized adults, assessing the prevalence of MetS, defined by an accepted definition (as mentioned below), b) studies conducted in adults aged ≥ 18 years, c) cross‐sectional study design or being the first phase of a longitudinal study (prospective follow-up studies), d) geographically and temporally defined population from any of the Asia-Pacific regional countries mentioned above, e) studies published in English or with detailed summaries and f) studies published before 30^th^ April 2016. Studies were excluded based on the following exclusion criteria: being confined to only a specific age/patient/community/ethnic group, being hospital/clinic-based, studies reporting the results of larger studies as duplications and studies conducted among Asians residing elsewhere. In addition during the final analysis most individual studies have excluded subjects with incomplete data for components of MetS.

Presence of ‘Metabolic Syndrome’ in the individual studies were considered only if defined according to one of the following accepted criteria; a) International Diabetes Federation (IDF) criteria, b) National Cholesterol Education Programme’s Adult Treatment Panel III criteria (NCEP/ATP III), c) modified IDF and modified NCEP/ATP III criteria with Asian cutoffs for Body Mass Index (BMI) and waist circumference, d) American Heart Association/National Heart, Lung and Blood Institute (AHA/NLHBI) criteria, or f) a harmonized criteria adopted at the time of the respective studies. The different definitions used in the respective studies are summarized in Table 1.

**Table 1:** Diagnostic criteria used in the different studies

Organization	Diagnostic Criteria
International Diabetes Federation (IDF)	Central obesity with waist circumference ≥ 90 cm for males and ≥ 80 cm for females, plus two or more of the following: 1. Systolic blood pressure ≥ 130 mmHg or diastolic blood pressure ≥ 85 mmHg or antihypertensive medication; 2. Fasting plasma glucose ≥ 5.6 mmol/L or previously diagnosed type 2 diabetes; 3. Plasma HDL cholesterol < 1.03 mmol/L for males and < 1.29 mmol/L for females; 4. Plasma triglycerides ≥ 1.7 mmol/L
National Cholesterol. Education Program, Adult Treatment Panel III (NCEP ATP III)	Three or more of the following: 1. waist circumference ≥ 102 cm for males and ≥ 88 cm for females; 2. systolic blood pressure ≥ 130 mmHg or diastolic blood pressure ≥ 85 mmHg or antihypertensive medication; 3. fasting plasma glucose ≥ 5.6 mmol/L or on medication for high blood glucose; 4. HDL cholesterol <1.03 mmol/L for males and <1.30 mmol/L for females; 5. triglycerides ≥ 1.7 mmol/L
Revised NCEP ATP III Criteria for Asians	Same as NCEP ATP III (above) with a modified waist circumference cut-off for Asians of ≥ 90 cm for males and ≥ 80 cm for females
An American Heart Association/National Heart, Lung, and Blood Institute (AHA/NHLBI)	Three or more of the following: 1. waist circumference ≥ 102 cm for males and ≥ 88 cm for females (for Asians ≥ 90 cm for males and ≥ 80 cm for females); 2. systolic blood pressure ≥ 130 mmHg or diastolic blood pressure ≥ 85 mmHg or antihypertensive medication; 3. fasting plasma glucose ≥ 5.6 mmol/L or on medication for high blood glucose; 4. HDL cholesterol <1.03 mmol/L for males and <1.30 mmol/L for females or on medications for reduced HDL cholesterol; 5. triglycerides ≥ 1.7 mmol/L or on medications for elevated triglycerides

### Data extraction and analysis

One reviewer was responsible for the extraction of data from the included articles, by using a standardized form. A second reviewer checked the accuracy of the data extracted. The following details were extracted from each study: a) details of the study (country/city, year of publication/year of survey and setting of the study), b) study methods (sample size, sampling method, age of the subjects in years and definition of MetS used in the study), and c) MetS prevalence data (for all adults, males and females). Any discrepancies in the data extracted in this manner were re-checked and resolved by discussion. A third reviewer was also involved where necessary.

Data not presented in the published manuscript (gender and area-specific prevalence) were obtained by contacting the corresponding author, or where possible calculated from the available data. When describing the prevalence of MetS for an individual country, the study that was the most recent, nationally representative or that had the highest sample size was selected. The age-standardized data is described here (unless stated otherwise), to enable for a meaningful comparisons between countries. When exploring the secular trends in prevalence of MetS in an individual country we only considered studies evaluating temporal changes between the same/similar populations or national surveys conducted at different times. As described above, only the age-standardized prevalence was considered when evaluating secular trends.

## Results

**Fig. 1:**
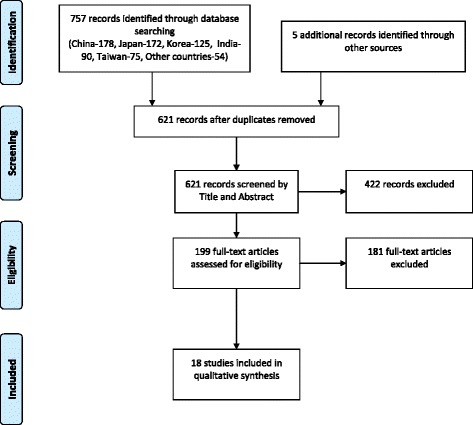
Summarized search strategy

This literature search yielded a total of 757 articles (China - 178, Japan - 172, South Korea - 125, India - 90, Taiwan - 75 and others – 54). Screening of reference lists of the included articles identified five additional articles. After removing duplicates, 621 articles remained. The title and abstract of these papers were screened to identify potentially relevant papers for full review. The full text was obtained for 199 papers deemed to be potentially relevant. From this total, 18 studies were eligible to be included in the final analysis. The summary of the search strategy is presented in Fig. 1. Of the 51 Asia-Pacific countries (WHO) we only located data for 15 counties (Australia, China, India, Indonesia, Japan, South Korea, Macau, Malaysia, Mongolia, Pakistan, Philippine, Singapore, Sri Lanka, Taiwan and Vietnam). The search revealed national surveys for 9 countries. Where national studies were not available the most recent regional study with the largest sample was selected (Australia, India, Indonesia, Japan, Pakistan and Vietnam), of which 4 studies were in urban settings and the remaining studies were in rural setting. Majority of the studies used a combination of definitions for MetS (*n* = 6), while five studies used the revised NCEP ATP III criteria and four studies used the IDF criteria. The remaining three studies used the NCEP ATP III criteria. The sample size ranged from 363 (an urban study in Pakistan) to 47325 (a national study in China). Majority of the studies included adults (>18 years in most and >20 years in few), without defining any upper limit for age, whilst in the remaining studies the included age group varied considerably.

### Prevalence of metabolic syndrome

The prevalence of MetS in the respective countries and the sample characteristics are summarized in Table 2. Almost all surveys reported prevalence data not only for all adults but also for males and females separately. There was wide between country variation in prevalence of MetS. A national survey from Philippines conducted in 2003 revealed the lowest reported prevalence of 11.9% according to NCEP ATP III criteria [17]. In contrast, the highest recorded prevalence in the region (49.0%) came from a study conducted in urban Pakistan (Karachchi, 2004) [18]. The next highest prevalence was reported from Malaysia (37.1%) (IDF criteria) [19]. The highest prevalence from a National survey was also reported from the same survey done in Malaysia in 2008 (37.1%, IDF criteria), in year 2008 [19]. The most recent prevalence was 31.3% (modified NCEP-ATP III), observed in a National survey conducted in South Korea in 2011–2012 [20]. Data from four National surveys were available in China, with the most recent survey (2009) reporting a prevalence of 21.3% (NCEP ATP III) [21].

**Table 2:** Prevalence of metabolic syndrome

Country	Study details (Study date; Study setting; Sample size; Age group)	Metabolic Syndrome definition	Prevalence of metabolic syndrome [95% CI]		
			All adults	Males	Females
Australia [22]	2004–2006; Rural; N – 1563; 25–74 years	IDF	35.8^ab^ [NR]	39.0^b^ [NR]	33.0^b^ [NR]
China	2009; National; N – 7488; > 18 years	NCEP ATP III	21.3 [20.4–22.2]	20.9 [19.5–22.2]	21.7 [20.4–23.0]
		IDF	18.2 [17.3–19.1]	16.2 [15.0–17.4]	20.0 [18.8–21.2]
India [23, 27]	2005; Rural; N – 4535; > 30 years	NCEP ATP III	24.6 [21.7–27.5]^b^	26.9 [24.3–32.9]^a^	18.4 [16.7–24.1]^a^
	2007; Urban; N – 2225; > 20 years	NCEP ATP III	16.9 [NR]	27.7^b^[NR]	43.2^b^ [NR]
		IDF	19.4 [NR]	31.8^b^ [NR]	47.2^b^ [NR]
Indonesia [42]	2006; Urban; N – 1591; 25–64 years	Modified NCEP ATP III	28.4^b^ [NR]	25.4^b^ [NR]	30.4^b^ [NR]
Japan [24]	2004; Urban; N – 2321; 40–87 years	NCEP ATP III	16.5^b^ [NR]	17.2^b^ [NR]	16.0^b^ [NR]
Macau [26]	2006; National; N – 1592; 18–44 years	IDF	NR	10.5 [NR]	3.7 [NR]
Malaysia [19]	2008; National; N – 4341; > 18 years	NCEP ATP III	34.3^b^ [32.9–35.8]	31.1^b^ [28.8–33.5]	36.1^b^ [34.3–37.9]
		IDF	37.1^b^ [35.6–38.5]	32.1^b^ [29.8–34.5]	39.8^b^ [38.0–41.6]
Mongolia [31]	2009; National; N – 1911 > 40 years	IDF	32.8^b^ [NR]	19.2^b^ [NR]	40.9^b^ [NR]
Pakistan [18]	2004; Urban; N – 363; > 25 years	IDF	34.8 [29.7–39.9]	31.8 [22.7–40.9]	36.1 [29.9–42.3]
		Modified NCEP ATP III	49.0 [43.8–54.1]	55.6 [46.5–64.7]	45.9 [39.7–52.1]
Philippine [17]	2003;National; N – 4753; > 20 years	NCEP ATP III	11.9 [10.6–13.2]	10.5 [8.8–12.2]	13.4 [11.6–15.2]
		IDF	14.5 [13.1–16.0]	11.8 [10.1–13.6]	17.4 [15.3–19.5]
Singapore [43]	1998; National; N – 4723; 18–69 years	IDF	20.2 [NR]	NR	NR
		AHA/NHLBI	26.9 [NR]	NR	NR
South Korea [20]	2011–2012; National; N – 9650; > 19 years	AHA/NLHBI	31.3^a^ [NR]	NR	NR
Sri Lanka [30]	2005–2006; National; N – 4485; > 18 years	IDF	24.3 [23.0–25.6]	18.4 [16.5–20.3]	28.3 [26.6–30.0]
Taiwan [33]	2005–2008; National; N – 2787; > 18 years	Modified NCEP ATP III	25.5 [NR]	25.5^ab^ [NR]	31.5^ab^ [NR]
Vietnam [44]	2011; Rural; N – 2443; 40–64 years	Modified NCEP ATP III	16.3 [14.0 − 18.6]	13.9 [11.5 − 16.2]	18.5 [16.3 − 20.7]

*CI* Confidence Interval, *NR* Not Reported

^a^Calculated using the available data; ^b^Crude Prevalence

Most studies reported a higher prevalence of MetS in females, except in studies conducted in rural Australia [22], rural India [23], urban Japan [24], urban Pakistan [18] and one National survey conducted in China [25] and Macau [26]. The highest prevalence in females (47.2%, IDF criteria) was observed in a study conducted in urban India [27], while the lowest prevalence (3.7%, IDF criteria) was observed in a National survey conducted in Macau [26]. A study conducted in urban Pakistan reported the highest prevalence of MetS in males (55.6%, modified NCEP ATP III criteria) [18]. The lowest prevalence in males was observed in two national surveys conducted in the Philippines (10.5%, NCEP ATP III criteria) and Macau (10.5%, IDF criteria) [17, 26]. An urban and rural comparison of MetS prevalence was available from five National surveys conducted in China, Malaysia, Mongolia and Sri Lanka [19, 28–31]. In all but the Mongolian study, urban adults had a higher prevalence of MetS, in comparison to the rural counterparts, irrespective of the MetS definition used (NCEP ATP III or IDF criteria) (Table 3).

**Table 3:** Urban and rural prevalence of metabolic syndrome

Country	Study details (Study date; Study setting; Sample size; Age group)	Metabolic Syndrome definition	Urban [95% CI]	Rural [95% CI]
China [28, 29]	2000–2001; National; N – 15540; 35–74 years	NCEP ATP III	18.6 [17.6–19.6]	12.7 [10.7–13.1]
	2007–2008; National; N – 47325 > 20 years	IDF	26.5 [NR]	22.1 [NR]
Malaysia [19]	2008; National; N – 4341; > 18 years	NCEP ATP III	36.0^a^ [33.9–38.0]	32.6^a^ [30.6–34.6]
		IDF	39.1^a^ [37.1–41.2]	35.0^a^ [33.0–37.1]
Mongolia [31]	2009; National; N – 1911 ≥ 40 years	IDF	31.7^a^ [NR]	34.1^a^ [NR]
Sri Lanka [30]	2005–2006; National; N – 4485; > 18 years	IDF	34.8 [31.8–37.9]	21.6 [20.2–23.0]

*CI* Confidence Interval, *NR* Not Reported

^a^Crude Prevalence

### Secular trends in prevalence

**Fig. 2:**
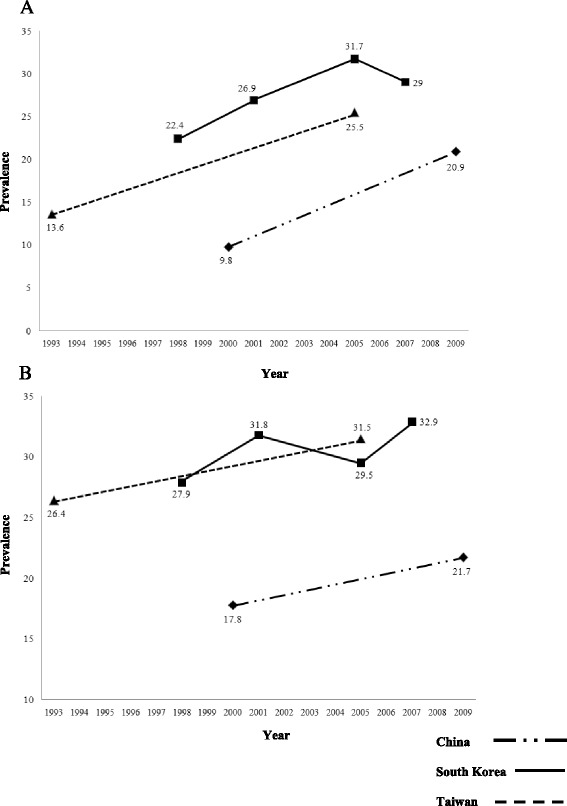
Secular trends in Prevalence in, **a**) Males and **b**) Females

Area of residence is a key factor determining the prevalence of MetS; therefore, when exploring secular trends in prevalence we only considered studies that evaluated the temporal change in prevalence between similar populations, prospective studies based on the same population or data resulting from National surveys conducted during different time periods. Such data were available for China, South Korea and Taiwan (Table 4) [21, 28, 32, 33]. An increase in the prevalence of MetS was observed in all three countries. The Korean National Health and Nutrition Examination Survey (KNHANES) survey reported a steady increase in the crude prevalence of the MetS in Korea from 24.9% in 1998 to 31.3% in 2007 according to the modified NCEP ATP III criteria [32]. A national survey in Taiwan using the modified NCEP/ATP III criteria also showed a significant increase in the prevalence of MetS from 13.6% (1993–1996) to 25.5% (2005–2008) over a period of 10–15 years [33]. A similar result was observed in China (13.7%, 2000–2001 and 21.3%, 2009) [21, 28]. These observations were also noted independently in males and females (Fig. 2).

**Table 4:** Secular trend in prevalence of metabolic syndrome

Country	Study details (Study date; Study setting; Sample size; Age group)	Metabolic Syndrome definition	Prevalence of Metabolic Syndrome [95% CI]		
			All adults	Males	Females
China [21, 28]	2000–2001; National; N – 15540; 35–74 years	NCEP ATP III	13.7 [12.9–14.5]	9.8 [9.0–10.6]	17.8 [16.6–19.0]
	2009; National; N – 7488; ≥ 18 years	NCEP ATP III	21.3 [20.4–22.2]	20.9 [19.5–22.2]	21.7 [20.4–23.0]
South Korea [32]	1998; National; N – 6907; >20 years	Modified NCEP ATP III	24.9 [23.7–26.1]^a^	22.4 [19.8–24.9]^a^	27.9 [25.5–30.2]^a^
	2001; National; N −4536; > 20 years	Modified NCEP ATP III	29.2 [27.8–30.5]^a^	26.9 [24.5–29.2]^a^	31.8 [29.1–34.5]^a^
	2005; National; N – 5373; > 20 years	Modified NCEP ATP III	30.4 [29.2–31.6]^a^	31.7 [28.8–34.6]^a^	29.5 [26.6–32.4]^a^
	2007; National; N – 2890; > 20 years	Modified NCEP ATP III	31.3 [30.1–32.5]^a^	29.0 [25.9–32.1]^a^	32.9 [29.4–36.4]^a^
Taiwan [33]	1993–1996; National; N – 2860; > 18 years	Modified NCEP ATP III	13.6 [NR]	13.6 [NR]	26.4 [NR]
	2005–2008; National; N – 2787; > 18 years	Modified NCEP ATP III	25.5 [NR]	25.5 [NR]	31.5 [NR]

*CI* Confidence Interval, *NR* Not Reported

^a^Calculated using the available data

## Discussion

This is the first comprehensive report to systematically evaluate the scientific literature on the prevalence and trends for MetS in the Asia-Pacific region. Prevalence, based on the most recent national surveys in the countries of the region ranged from 11.9% in Philippines (2003) to 37.1% in Malaysia (2008). In most countries nearly 1/5^th^ of the adult population or more were affected by MetS, comparable with the IDF estimation that nearly one quarter of the world’s adult population is suffering from MetS [34]. Data are comparable with other regions of the world (Table 5) and indicates a secular increase in prevalence in the region. Despite differences in methodology, diagnostic criteria and age of subjects studied, the Asia-Pacific region is facing a significant epidemic of MetS. Hence strategies aimed at primary prevention are required to ameliorate a further increase in the epidemic and for the reduction of the morbidity and mortality associated with MetS.

**Table 5:** Prevalence of metabolic syndrome in the different geographical regions

Region	Year of Publication	Prevalence of Metabolic Syndrome (%)
Asia-Pacific	Present study	11.9–37.1
Africa [45]	2012	12.5–62.5
Central America [46]	2015	23.0–35.1
Europe [47]	2014	11.6–26.3
Middle East [48]	2012	13.6–36.3
South America [49]	2011	18.8–43.3
South Asia [50]	2016	26.1

The Asia-Pacific region is home to more than half of the world’s population and includes some of the world’s richest alongside some of the poorest and least developed countries [35]. Despite persistent levels of underweight in some countries, overweight, obesity and associated non-communicable diseases have become a major public health concern for the entire region [35]. The increased prevalence of MetS in the region could be attributed to regional changes in disease patterns from communicable to non-communicable diseases, resulting from increased life expectancy, rapid population growth and unplanned urbanization [36]. This ‘epidemiological transition’ is closely linked to the rapid industrialization occurring in the region, as evidenced by the higher prevalence of MetS observed among urban residents. Unhealthy lifestyle changes associated with urbanization such as physical inactivity, changes in diet and stress, are likely associated with increased risk of MetS. Rural-to-urban migration is also known to be a major factor in the epidemiology of non-communicable diseases, including diabetes and obesity [37]. Migrants typically change their lifestyles considerably within a short period of time and physical activity status quickly reaches urban levels with acquisition of a metabolic risk similar to that of urban dwellers [37]. Our results also show that the prevalence of MetS is also rising in rural communities of the Asia-Pacific. Increased mechanization of the agriculture industry, automation of daily activities, popularization of television and increased computer usage in rural areas are leading to changes in lifestyle with resultant decreases in physical activity [38].

Parallel to the increase in MetS and obesity, the prevalence of associated non-communicable diseases such as diabetes is also rising in the region. It is estimated that the region has more than 138 million people with diabetes, a number which is expected to rise to 200 million by 2035 [39]. Hence, the MetS epidemic and associated non-communicable diseases are a significant public health concern in the region at present. It is important to look at shared risk factors in order to develop harmonized universal preventive strategies, through existing regional collaborations. Physical inactivity/sedentary life style, urban residency, smoking, alcohol consumption, family income and level of education are some of the modifiable factors identified as associated with MetS from the studies included in the present review. Increasing age, female gender and family history of diabetes were some of the other non-modifiable factors associated with the presence of MetS. Due to the limited number of studies or due to variations in definitions/classifications of risk factors between studies a meta-analysis and quantification of the strength of association could not be performed.

However, of the 51 countries in the Asia-Pacific region, data on MetS was available for only 15 countries. Future, well-designed epidemiological studies with representative population samples would provide the basis for a better understanding of the extent and public health implications of MetS in the region. Such studies would also help to develop standardized measurement criteria and definitions of MetS, to allow meaningful comparisons within and between populations. Furthermore, the actual health care costs and economic burden of MetS in the Asia-Pacific region have not been assessed to date. It is necessary to conduct future studies to assess the direct and indirect costs of MetS in countries of the region. Such studies need to focus on standardized calculation methods to make meaningful between country comparisons. Future research also needs to focus on identification of shared risk factors for MetS. However, as highlighted previously such studies need to be conducted in a prospective sample, using standard definitions for risk factors. Regional organizations such as the WHO could play an important role in introducing such assessment standards for future research conducted in the region.

The strengths of the current systematic review are the comprehensive and easily replicable search strategy applied and the selection of studies through the application of well-defined inclusion/exclusion criteria. We would also like to highlight several limitations in the present review. There was no uniformity of MetS definition, age groups, waist circumference cut-offs, and study settings in the studies included in the present review, resulting in limitations in comparability. There were four definitions of MetS primarily used in the studies included in the present analysis (IDF criteria, NCEP ATP III, revised NCEP ATP III and AHA/NLHBI). The main difference between the IDF criteria and the other 3 criteria is that the IDF requires the presence of abdominal obesity (defined by waist circumference) as an essential factor for defining MetS, whilst in the other criteria it is not an essential requirement [40]. Since, Asians are known to develop metabolic complications at lower thresholds for abdominal obesity most criteria separately define central obesity cut-offs for Asians and other populations. This difference has been taken into account by most studies included in the present analysis. However, even in Westerners in the NCEP ATP III criteria, a difference of 14 cm in current abdominal obesity criteria across genders may be debatable, leading to dilution of MetS in women or a failure of encompassing men with MetS at increased cardio-metabolic risk [41]. It is very well known that the prevalence of MetS varies and depends on the criteria used in different definitions, as well as the composition (sex, age, race and ethnicity) of the population studied. Hence, the differences in the definitions used in the studies included in the present analysis makes it difficult for meaningful comparisons between countries. However, it is generally accepted that no matter which criteria are used, the prevalence of MetS is high and rising in most counties as a result of the obesity epidemic [41]. Furthermore, most studies included in the present analysis are nearly 5–10 years old, hence the current prevalence of MetS in the region and the associated disease burden is likely to be much higher. For example, the most recent national prevalence study in South Korea approximately 5 years ago observed a prevalence of 31.3%. Countries for which data on secular trends are available have shown an increase in prevalence of about 50–75% over a period of 10 years, highlighting the need for newer well-designed epidemiological studies to identify the most recent extent of the MetS epidemic in the Asia-Pacific region.

## Conclusions

Despite differences in methodology, diagnostic criteria and age of subjects studied, the Asia-Pacific region is facing a significant epidemic of Metabolic Syndrome. In most countries nearly 1/5th of the adult population or more were affected by Metabolic Syndrome with a secular increase in prevalence. Hence regional strategies through existing collaborative partnerships aimed at primary prevention are required to ameliorate a further increase in the epidemic and for the reduction of the morbidity and mortality associated with Metabolic Syndrome.

## Additional file

Additional file 1: PRISMA 2009 Checklist. (DOC 63 kb)

## Abbreviations

AHA/NLHBI: American Heart Association/National Heart, Lung and Blood Institute
BMI: Body mass index
IDF: International diabetes federation
MetS: Metabolic syndrome
NCEP ATP III: National cholesterol education program adult treatment panel III
WHO: World Health Organization
